# After-meal blood glucose level prediction for type-2 diabetic patients

**DOI:** 10.1016/j.heliyon.2024.e28855

**Published:** 2024-04-02

**Authors:** Benzir Md Ahmed, Mohammed Eunus Ali, Mohammad Mehedy Masud, Mohammad Raihan Azad, Mahmuda Naznin

**Affiliations:** a Department of Computer Science and Engineering, Bangladesh University of Engineering and Technology, Dhaka, 1000, Bangladesh; b College of IT, United Arab Emirates University, Al Ain, 15551, United Arab Emirates; c Department of Computer Science and Engineering, United International University, Dhaka, 1212, Bangladesh; d Physicians East-Endocrinology, Greenville, 27834, USA

**Keywords:** After-meal glucose, Blood glucose prediction, Machine learning, Type 2 diabetes

## Abstract

Type 2 Diabetes, a metabolic disorder disease, is becoming a fast growing health crisis worldwide. It reduces the quality of life, and increases mortality and health care costs unless managed well. After-meal blood glucose level measure is considered as one of the most fundamental and well-recognized steps in managing Type 2 diabetes as it guides a user to make better plans of their diet and thus control the diabetes well. In this paper, we propose a data-driven approach to predict the 2 h after meal blood glucose level from the previous discrete blood glucose readings, meal, exercise, medication, & profile information of Type 2 diabetes patients. To the best of our knowledge, this is the first attempt to use discrete blood glucose readings for 2 h after meal blood glucose level prediction using data-driven models. In this study, we have collected data from five prediabetic and diabetic patients in free living conditions for six months. We have presented comparative experimental study using different popular machine learning models including support vector regression, random forest, and extreme gradient boosting, and two deep layer techniques: multilayer perceptron, and convolutional neural network. We present also the impact of different features in blood glucose level prediction, where we observe that meal has some modest and medication has a good influence on blood glucose level.

## 1 Introduction

According to *Diabetes Atlas* 2021 [1], *Diabetes*, a chronic metabolic disorder disease, is the fastest growing health emergencies in the twenty-first century. In 2021, approximately 537 million adults (20–79 years) were living with diabetes, and nearly 7 million diabetes related death had been reported. The number of people with diabetes is projected to rise to 643 million by 2030 and 783 million by 2045. Thus, researchers, health workers, and policy makers take different initiatives to combat diabetes. As this disease cannot be fully cured, if the disease is not controlled then a diabetic patient will have long-term diabetes related complications such as cardiovascular disease, kidney failure, diabetic retinopathy, etc. Thus, one of the key focuses of the practitioners in this area is to manage diabetes through a controlled life-style (e.g., Ref. [2]).

The main goal of a diabetes management system is to keep the fasting *Blood Glucose Level* (BGL) within the euglycemic (normal) range of 70 mg/dL and 126 mg/dL before meals and under 180 mg/dL 2 h after meals. Meal-intake has a direct influence on BGL, in particular, the nutrient Carbohydrate (CHO) (or simply carb) in a meal increases the BGL [3], and protein, fat, & fiber part of the meal slow down the meal consumption process. Hence, the nutritional ingredients of a meal play an important role in determining the BGL. Other factors that influence the BGL include *insulin* or medicine, physical exercise, etc. Insulin or diabetes medicine reduces the BGL to offset the glucose hike due to meal intake. On the other hand, physical exercise reduces BGL by burning energy. In this research, we propose a machine-learning based system to predict the 2 h after meal BGL of Type 2 diabetes patient, taking the meal intake, insulin or medicine, physical exercises, etc. into account.

There are a number of key benefits for predicting BGL 2 h after a meal. If we could predict the BGL 2 h after a meal before eating that meal, a diabetic patient could adjust his/her diet. Also s/he could adjust the intensity and duration of his/her physical exercise, and could adjust his/her insulin dosage amount accordingly to have a euglycemic state 2 h after meal. It is important to note that knowing 2 h after meal BGL also helps patients avoid life threatening abnormal conditions, such as hyperglycemia and hypoglycemia. It should be noted that hyperglycemia is a condition when the BGL is above 126 mg/dL during fasting and above 180 mg/dL 2 h after a meal, and hypoglycemia is a condition when the BGL drops below 70 mg/dL.

With growing urgency to control diabetes, there have been growing research efforts in the BGL prediction area for both Type 1 Diabetes Mellitus (T1DM) and Type 2 DM (T2DM) diabetes [4]. Most of the existing research focus on BGL prediction for T1DM diabetes (the type of diabetes due to autoimmune system disorder) (e.g., Refs. [[5], [6], [7]]). Many T1DM patients use *Continuous Glucose Monitoring* (CGM) devices to track BGLs. CGM devices provide a BGL reading every 5 min totaling 288 readings per day. This high frequency data generation motivates the application of data-driven techniques including Deep Learning (DL) based predictive models (e.g., Refs. [[7], [8], [9], [10], [11]]). Data collected using CGM devices are time-series data. On the contrary, T2DM patients usually measure BGL values using a finger stick test with a personal blood glucose meter. Data collected using finger stick tests are discrete data. At the upper end, a T2DM patient may have one or two data points a day. Hence, the time-series models that are developed using CGM time-series data of T1DM patients cannot be readily used to predict BGL for T2DM patients.

Few research works focus on BGL prediction for T2DM patients [9,[12], [13], [14], [15]]. Faruqui et al. [9] have collected 6 months of data of 10 T2DM patients to forecast next day BGL using deep learning models. Karim et al. [16] have collected three weeks of four T2DM and one T1DM patients' data to use as input to a *Neural Network* model. Deng et al. [17] have collected CGM data from 40 T2DM patients. Their best performing model used the publicly available OhioT1DM [18] dataset. Gyuk et al. [13] have collected CGM, meal, insulin, and medication data of 26 T2DM patients for three weeks. Yang et al. [14] have collected CGM readings of 100 T1DM and T2DM patients for 4 days. Most of the above mentioned works [[12], [13], [14]] have collected T2DM patients’ CGM data.

Faruqui et al. in Ref. [9] and Albers et al. in Ref. [15] are the only two research works that have collected discrete T2DM data from glucometers. Faruqui et al. [9] have treated the daily glucose data as time series data. Each day glucose value is used as one data point. Hence, their focus is to predict the next day BGL, which is a different goal from ours. Albers et al. [15] have used two physiological models to show that even with sparse glucometer data it is possible to produce well enough personalized models.

The key challenges of predicting 2 h after meal BGL prediction is the scarcity of data that captures different factors affecting the diabetes in free living conditions. Also, there are a large number of factors affecting the 2 h after meal blood glucose, which may vary person to person.

To overcome the above challenges, in this study, we have collected discrete fingertip BGL values, diabetes medication or insulin dosage, physical activity, food value of meal intake, demographic in-formation, and clinical profile of five T2DM patients in free living condition for six months. We have implemented a smart device app to collect those data. We have annotated the meal intake data with appropriate nutrition food value with the help of nutrition experts. There were 23 features in the model ready dataset. Finally, we present that the data-driven models can forecast 2-hr postprandial BGLs in T2DM patients leveraging discrete BGL readings, diabetes medication doses, physical activity details, meal nutritional values, and profile information with a Root Mean Square Error (RMSE) of 31.87 mg/dL, which is an acceptable error for real-life applications of BGL predictions [16,19,20].

We list the major contributions of our research as follows.

• We have prepared a comprehensive dataset consisting of 900+ events collected over six months from 5 T2DM patients in free living condition.
• We have collected the meal image (then convert these into corresponding food nutritional values), information about exercise, medicine, etc. for each collection event as these are the primary factors influencing the BGL.
• We have provided an effective proof of concept of a novel comprehensive BGL prediction frame-work. In this framework, a data preprocessing step is implemented to ensure data quality, importance of different features are identified, influence of feature combinations are examined, and finally the first data driven system to predict 2 h after meal BGL values from discrete fingertip BGL and other values of T2DM patients with the state-of-the-art prediction performance is presented.
• We have presented a comprehensive evaluation of our proposed system with a number of traditional machine learning (ML) and deep learning (DL) techniques, and a detailed analysis of the results.

The remaining sections of the paper are organized as follows. Section 2 reviews the existing literature on BGLP. Section 3 discusses the BGLP framework, data collection process, data preprocessing, and the ML techniques that have been used in this research. Section 4 outlines the experiments that are conducted, the evaluation metrics that are used, and the hyperparameters that are selected. Section 5 discusses the results of the experiments. Section 6 presents the discussion, the limitations of this study, and the potential avenues for future research. Finally, Section 7 provides the summary of the research.

## 2 Related works

Most of the existing research have focused on BGL prediction that have reported performance results on 30-min Prediction Horizon (PH), quite a few on 60-min PH, and only a few on 2-hr PH. For a diabetic patient, the *fasting BGL* and the *2 h after meal BGL* are two important metrics that are usually observed to manage diabetes well all over the world [21]. Due to its high importance, we focus on 2 h after meal BGL prediction, i.e. the 2-hr PH. We will use the *Root Mean Square Error* (RMSE), a statistical regression metric that provides the precision level as given in Equation.

2 and *Clarke's Error Grid Analysis* (CEG) [22], a clinical regression metric that provides the clinical applicability level for model performance comparison. CEG has five regions: A, B, C, D, and E. Higher values in the A and B regions mean more correct predictions and are better. Lower values for C, D, and E regions mean less incorrect predictions and are better.

### 2.1 BGL prediction of type 1 diabetes patients

It is observed in the literature that the history of BGLs is very effective in predicting future BGLs. Therefore, it is the mostly used input feature in BGL prediction. Though over 90% of the diabetic patients have Type 2 diabetes, majority of the research use time-series of CGM readings of T1DM patients as observed from Table 1. This is mostly due to the high volume of data a CGM device can produce per day. Three types of BGL prediction models: *Physiological models*, *Data Driven models*, and *Hybrid models* are mostly used by the researchers for BGL prediction of T1DM patients. We now discuss in details as follows.

**Table 1:** BGL prediction performances of the existing literature in terms of RMSE and CEG (of A + B regions combined). (**Real data**). Metric CEG has a % sign. Notations: Glucose (G), Insulin (I), Meal (M), Exercise (E), Physiological (Physio.), Genetic Programming (GP), Grammatical Evolution (GE), Markov Chain Monte Carlo (MCMC), Ridge Regression (RR), Differential Evolution (DE), Performances (Perf.), Data Assimilation (DA).

Ref	Year	Input Fea- tures	Dataset	Modeling Technique	Perf. $\frac{RM\;SE}{C\;EG\left(\%\right)}$	
					PH = 30	PH = 120
**Physiological Models**						
[23]	2015	G	12 T1DM, 10 days	Physio.	98.00%	87.00%
[20]	2019	G, I, M	10 T1DM, 2 weeks	Physio.	17.67	40.46
					99.05%	95.27%
**Traditional ML Models**						
[25]	2015	G, I, E, M	15 T1DM, 5–22 days000	Extreme Learning Machine	6.10	–
[11]	2015	G,ΔG, E	10 T1DM, 6 days	SOM	11.42	31.00
					93.84%	82.37%
[32]	2017	G, I, M	1 T1DM, 46 days	GE, GP, MCMC	–	99.56%
[33]	2017	G	17 T1DM, 5.73 days	Regime-switching AR	5.83	–
[26]	2018	G	12 T1DM, 14 days	SVR, DE	10.78	–
[34]	2019	G,I,M	5 T1DM, 15 days	Markov, GE, RF, Ensem- ble	98.84%	94.17%

Ref	Year	Input Fea- tures	Dataset	Modeling Technique	Perf. $\frac{RM\;SE}{C\;EG\left(\%\right)}$	
					PH = 30	PH = 120
[19]	2020	G, I, M	24 T1DM, 37.8 days	Physiologically-informed FNN	–	38.00
					–	94.00%
[35]	2020	G, I, E, M	6 T1DM, 8 weeks	ARX, RR	19.48	–
[36]	2021	G	225 T1DM, 6 months	ANN, penalty	21.63	–
					99.72%	–
**Deep Learning Models**						
[37]	2017	G, I, M	10 T1DM, 400 days	LSTM	21.4	–
[6]	2018	G	6 T1DM, 8 weeks	LSTM, Physio. loss func- tion	20.10	–
[38]	2019	G, I, E, M	10 T1DM, 6 weeks	CNN	21.07	–
[8]	2019	G	451 T1DM, 398578 samples	LSTM	5.93	–
					99.94%	–
[9]	2019	G, E, M	10 T2DM, 6 months	LSTM, Transfer learning	99.34%	–
[28]	2020	G	12 T1DM children, 7 days	MLP	6.31	–
[10]	2020	G, I, M	9 TIDM, 4 days	Stacked LSTM	6.42	–
[31]	2020	G, I, M	34 T1DM, 102–268 days	CNN, LSTM	9.18	–
[39]	2020	G	20 T1DM, 878k Data points	Stacked LSTM	11.96	–
[40]	2020	G, I, E, M	6 T1DM, 8 Weeks	Dilated RNN	18.9	–
[17]	2021	G	40 T2DM	CNN	19.08	–
[41]	2022	G, I, M	12 T1DM, 8 Weeks	Attention-based BiRNN	18.64	–
[42]	2022	G, I, M, time	12 T1DM, 8 Weeks	Channel DL	18.93	–
**Hybrid: Physiological + Data-driven Models**						
[43]	2012	G, I, E,M	15 T1DM, 5–22 days	Physio. and SVR	6.03	7.62
[44]	2012	G, I	15 T1DM, 7 days	Linear model, Meal Ab- sorption Model, FNN	14.00	–
[15]	2017	G	3 T2DM, 23 days (539 readings)	Physio., Gaussian, DA	–	23.87
[16]	2020	G, I, M	4 T2DM, 1 T1DM, 3 weeks	Physio., FNN	–	31.50
					–	98.13%

#### 2.1.1 Physiological models

Among BGL prediction models, *physiological models* are the least used due to the complexity and uniqueness of individual biological systems, and it needs expert domain knowledge. Among the research works [20,23] that have used physiological models, the work by Liu et al. [20] has performed the best with RMSE of 40.46 mg/dL and a 95.27% correct prediction in the A + B regions for a 2-hr PH. Their compartmental composite model of glucose-insulin dynamics uses a deconvolution technique on the CGM values.

#### 2.1.2 Data-driven models

Majority of the research efforts have used data-driven models: traditional Machine Learning (ML) models and Deep Learning (DL) models. Frandes et al. [24] have used 4–7 days of CGM readings and time of different events of 17 T1DM patients as input on their proposed nonlinear Auto-Regressive Neural Network (AR-NN). They have achieved an exceptional performance with an RMSE of 2.37 mg/dL for 30-min PH. Georga et al. in Ref. [25] have investigated the use of *Extreme Learning Machines* which are in plain eyes Feedforward Neural Networks (FNN) on 5–22 days of glucose, insulin, exercise, and meal data of 15 T1DM patients. They have achieved an RMSE value of 6.10 mg/dL for.

30-min PH. There are other works that have used FNN, Self-Organizing Map (SOM), Wavelet Fuzzy NN (WFNN), Linear Regression (LR) [11], Recursive least squares (RLS), Support Vector Regression (SVR) with differential evolution [26], Random Forest (RF) [27] and have achieved RMSEs of 11.42 mg/dL (for 30-min PH) & 31.00 mg/dL (for 2-hr PH), 8.93 mg/dL (for 30-min PH), 10.78 mg/dL (for.

30-min PH), and 14.63 mg/dL (for 30-min PH), respectively.

Since more than a decade, *Deep Learning* (DL) techniques have been used in a great number of research works including BGL prediction. Alfian et al. [28] have used 7 days of CGM readings of 12.

T1DM children and eight statistical features as input to the MLP model with two hidden layers of.

100 neurons each. They have achieved a good performance with an RMSE of 6.31 mg/dL for 30-min.

PH. They have shown that the addition of the statistical features have enhanced performance.

Another group [10] have used 4 days of glucose, insulin (both basal and bolus), and meal data of 9 T1DM patients from the D1namo dataset [29] as input. They have applied a different Long Short-Term Memory (LSTM) based Recurrent Neural Network (RNN) [30] layer for each of the four input features separately followed by a dense layer. All the outputs were then concatenated to get the final output. This model has achieved an RMSE of 6.42 mg/dL for 30-min PH. Gu et al. [31] have taken the same approach except that they have used CNN on individual features, concatenated the outputs, and finally have used the LSTM model to get the final output. They have used their model on a publicly available dataset on tidepool.org that has 102–268 days of glucose, insulin, and meal data of 34 T1DM patients and received an RMSE of 9.18 mg/dL for 30-min PH.

Aliberti et al. [8] have used LSTM model on a large dataset of 451 T1DM patients and have received an average RMSE value of 5.93 mg/dL for 30-min PH. Wang et al. [39] have used a model with stacked LSTM layers on 878k data points of twenty T1DM patients and received an RMSE of 11.96 mg/dL for 30-min PH. Zhu et al. [40] have used *Dilated Recurrent Neural Network*s (DRNN) with vanilla RNN cells on the OhioT1DM dataset and received an RMSE of 18.9 mg/dL on 30-min.

PH. In another work, Zhu et al. [41] have used an attention-based bidirectional RNN (BiRNN) model with confidence provided by *evidential regression* on 8 weeks of CGM, insulin, and meal data of 12.

T1DM patients to compute personalized BGL prediction and have received an improved RMSE of.

18.64 mg/dL. Yang et al. [42] have proposed an autonomous channel DL framework with adaptive input selection capability on the same dataset for BGL prediction. It appears that SVR, MLP, CNN, and LSTMs are being frequently used by the researchers with good performance.

#### 2.1.3 Hybrid models

Among the hybrid models, the work by Georga et al. [43] have used insulin absorption, kinetics, and dynamics model to simulate plasma insulin concentration and glucose absorption model to simulate glucose plasma appearance rate. In addition to these, hour of the day, and activity energy are used as input features to the SVR BGL prediction model. Their best prediction model yields a state of the art performance with a RMSE value of 6.03 mg/dL for 30-min PH and 7.62 mg/dL for 2-hr PH. Another work by Karim et al. [16] have used the glucose absorption curve from a physiological model [45], insulin dosage amount, and CGM readings as input to a FNN with 20 hidden layers and achieved an RMSE of 31.5 mg/dL for 2-hr PH. In another follow-up work in Ref. [46], in addition to the previous inputs the researchers have added the total area under the absorption curve, and basal insulin dosage time, and have found an improved RMSE of 30.92 mg/dL.

### 2.2 BGL prediction of type 2 diabetes patients

Only a few [9,[13], [14], [15], [16]] of the relevant research on BGL prediction have used T2DM patients' data that we already have mentioned in Section 1. Among these works, all except [9,15] have used CGM readings of T2DM patients. Faruqui et al. [9] have used discrete BGLs of T2DM patients. However, they have used that data to predict the next day's BGL. The research work by Albers et al. [15] is the only attempt where they have used physiological models to predict 2 h after meal BGLs using discrete BGLs of T2DM patients. Hence, we observe that the majority of the previous studies have used CGM data. There is a lack of research effort using the discrete BGL values of T2DM patients. Therefore, in this work we have proposed a data-driven system to predict BGL values 2 h after meal from discrete fingertip BGL and other values of T2DM patients.

## 3 BGL prediction framework

**Fig. 1:**
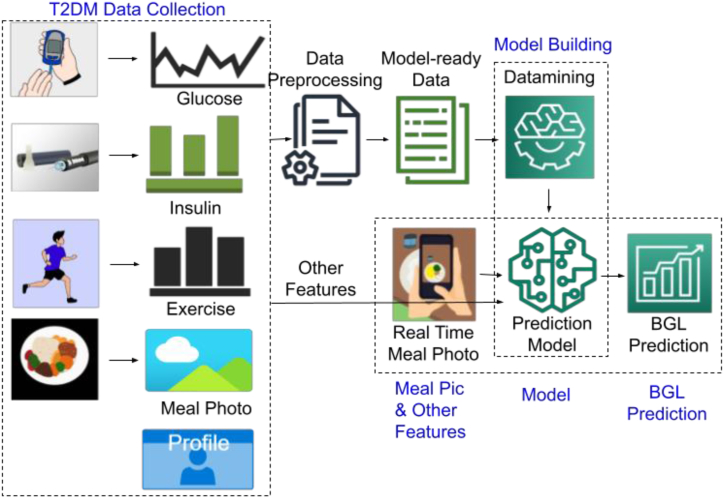
Bgl prediction framework.

Our proposed BGL prediction framework is presented in Fig. 1. The data collection process is the first and the most important task of this work. The data collection process is described in Subsection 3.1. Data is collected from five (5) T2DM patients in free living condition. The collected data is usually in raw form. They need to be preprocessed before they can be used as input data by any model. The data preprocess step, described in Subsection 3.2 provides the *model ready dataset*. We have conducted experiments utilizing Principal Component Analysis (PCA), a feature selection strategy, to explore its potential contributions. The model ready dataset is used to train and test the potential models to find the optimized prediction model. The models are described in Subsection 3.4. The optimized model will be loaded to a smart device. When the user takes a meal image just before eating a meal, the loaded model will use that and other concurrently collected features to predict the BGL after 2 h of meal intake. This will help a diabetic patient to adjust her diet or lifestyle instantaneously.

**Table:** 

**Algorithm 1** BGL Prediction Framework Training (*X*)	
**Input:***X* = *{x*1*, x*2 *, …, xn }*: T2DM Patient Data	
**Output:** M: Trained Model	
1: *Xm ←* handleMissingValues(*X*)	*▷* Section 3.2
2: *Xn ←* categoricalToNumeric(*Xm*)	*▷* Section 3.2
3: *Xr ←* normalize(*Xn*)	*▷* Section 3.2
4: *Xt ←* PCA(*Xr*)	*▷* Section 3.3
5: *M ←* BuildModel(*Xt*)	*▷* Algorithm 2
6: Return M

**Table:** 

**Algorithm 2** BuildModel (*X*)
**Input:***X* = *{x*1*, x*2 *, …, xn }*: Preprocessed T2DM Patient Data
**Output:** M: Trained Model
1: *P ←* getModelParameters()
2: *M ←* initializeModel(*P*)
3: RMSE *←* modelCompileAndFitAndPredict(*M*, *X*)
4: *M ←* OptimizeModelHyperParameters(*M*,RMSE)
5: Return M

**Table:** 

**Algorithm 3** BGL Prediction Framework Deployment (*M*)	
**Input:***M*: Trained model (from algorithm 1)	
**Output:** y: Predicted BGL 2h after meal	
1: *X ←* get Meal Photo and other data before meal	*▷* See Fig. 1
2: *y ←* Predict(*M*,*X*)	*▷* Prediction by model
3: Return y

### 3.1 Data collection process

In this subsection, we discuss the details of the data collection process using the *DiabetesPal* smart app designed by us. The user profiles, used glucometers, etc. are also discussed.

#### 3.1.1 User profiles

In this step, the data has been collected for six months from five Type 2 diabetic and prediabetic patients in the range of 46–82 years of age in free living conditions. *Written Informed Consents* have been taken from all the patients as a standard practice of data collection. Appropriate approval related to data collection involving human subjects is taken from Bangladesh University of Engineering and Technology (BUET), reference no: Estt./Ta-4/140548/R-3617, dated 04/12/22. User profiles are given in Table 2. Among them, three are male patients and two are female patients. They have various lengths of diabetes history. The patients have taken a blood glucose reading just before a meal, and another reading after 2 h of the meal intake. They have used two different glucometers: Accu- Chek Performa, and One Touch Verio Flex. The patients have taken images of the meal using their personal smartphone. They have taken this data for the same meal of the day that is convenient for them for six months living a normal life at home.

**Table 2:** User profiles of the dataset.

Id	Gender	Height (cm)	Weight (kg)	Family History	Duration (yrs)	Age
7	Male	160.02	54.50	No	22	82
9	Female	154.94	40.00	Yes	16	72
10	Male	160.02	66.00	Yes	22	44
11	Female	157.48	59.00	Yes	22	53
12	Male	172.72	74.10	Yes	22	52

#### 3.1.2 Data collection using the “DiabetesPal” smart app

**Fig. 2:**
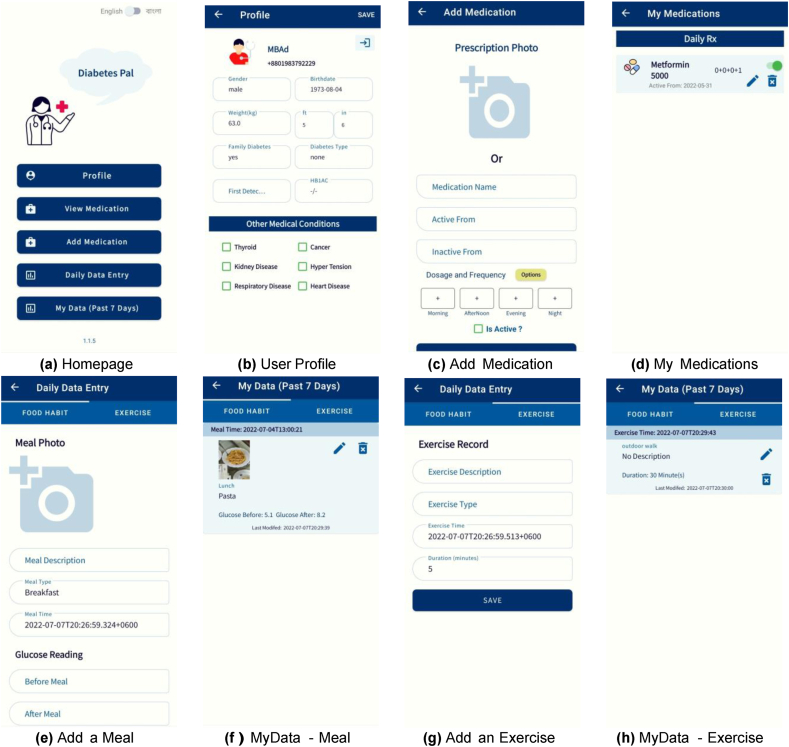
Our DiabetesPal smart app screenshots: (a) Homepage, (b) User Profile, (c) Add Medication, (d) My Medications, (e) Add a Meal, (f) MyData - Meal, (g) Add an Exercise, (h) MyData - Exercise.

A smartphone app “DiabetesPal” has been developed (shown in Fig. 2). The patients have used the app to record meal image, two BGL readings: one just before and one 2 h after meal, and all the physical activities data each day. For physical activity, they have entered the activity type, time, and duration. The patients are initially required to create an account on the app. At that account creation or at a later time, the patients record diabetes oral medication or insulin dosage, clinical profiles, and demographic information. Profile and medication information are editable in the app throughout the data collection step.

### 3.2 Data preprocessing

Daily data is directly uploaded to the cloud for storage from the app. All this data has gone through data preprocessing. All of the meal images are manually inspected by a nutrition expert to estimate the nutritional values. For each meal image, the serving size of each ingredient is eye estimated. The Carbohydrate (CHO), protein, fat, fiber, and calories information per serving of each ingredient is taken from Refs. [47,48]. The Glycemic Index (GI) information has been taken from Ref. [49]. The total CHO, protein, fat, fiber, and calorie of each meal is found by adding the values of all the ingredients. Glycemic Load (GL) is used in place of GI. GL also considers the amount of the ingredient in grams along with its GI [50]. To find the Glycemic Load (GL) value Equation (1) given below is used. Moreover, the nutrition expert has ensured that the differences among the macronutrient composition of the meals for different patients in the free-living conditions are negligible.

(1) $$\text{GL}=\frac{\text{GI}.\left(CHO\;amount\;in\;gm\right)}{100}$$

Patients have manually recorded all information related to physical activities like exercise time, type, and duration in the app (shown in Fig. 2). Physical activities can lower BGL as long as 10 to 24 h after the activity by making body cells sensitive to insulin [51]. Hence, any exercise within the 24-hr window before the 2 h BGL reading after meal can be considered. To include the more considerable effect, we have used a shorter 6-hr window. In our experiments we have included only the immediate exercise data within the 6-hr window. We have used the duration of exercise in minutes as the only exercise feature. In Ref. [19] by Kushner et al., they have used intermittent heart rate and step count data. They have found that these data do not have significant influence on BGLs. Xie et al. [35] have demonstrated that there is no added benefit for adding heart rate as a feature. Yet, we have plans to collect and include the step count, calories burned, heart rate, and quality of sleep as input features to further investigate their influence on future BGLs in our future work.

Patients have manually recorded their medication information: name of medicine, dosage amount, and frequency every time their prescriptions change. Any diabetes medicine taken by the patients can be categorized into the following nine categories: Metformin, Sulfonylurea, Pioglitazone, DPP-4- Inhibitor, GLP-1-RA, SGLT2-Inhibitor, Basal Insulin, Prandial Insulin, and Mixed Insulin [52]. From these categories we get nine features for our models. The values of the features are the dosage amounts.

After extraction of data from the source, we get six (6) demographic features: *Gender, Height, Weight, Family History, Duration*, and *Age*; one (1) exercise feature: duration; nine (9) diabetes medicine features: Metformin, Sulfonylurea, Pioglitazone, DPP-4-Inhibitor, GLP-1-RA, SGLT2-Inhibitor, BasalInsulin, PrandialInsulin, and MixedInsulin; six (6) meal features: CHO, Protein, Fat, Fiber, Calorie, and GL; and one (1) BGL reading feature: BGBefore. In total we have 23 features. One sample data is given in Table 3.

**Table 3:** One sample data of input features.

**Demographic**		**Exercise**		**Medicine**		**Meal**		**BGL**	
**Feature**	**Value**	**Feature**	**Value**	**Feature**	**Value**	**Feature**	**Value**	**Feature**	**Value**
Gender	Male	Duration	30	Metformin	500	CHO	32	BGBefore	54.5
Height	154			Sulfonylurea	80	Protein	6		
Weight	40			Pioglitazone	0	Fat	5		
Family	Yes			DPP-4-	0	Fiber	3		
History				Inhibitor					
Duration	16			GLP-1-RA	0	Calorie	203		
Age	72			SGLT2-	0	GL	101		
				Inhibitor					
				BasalInsulin	10				
				PrandialInsulin	12				
				MixedInsulin	24				

Through frequent communication with the subjects, we have observed that over 95% of the cases they have adhered to medication. We have identified the rest of the 5% cases and have collected the correct dosage amount. We have also observed that they haven't missed to report their physical activity information. Any sample data with missing meal image, and missing BGL reading (before or after) is removed. Categorical features are converted to numerical features with one-hot encoding. All of the other numerical features are normalized with the *min-max normalization* techniques to avoid biases from features with large values.

### 3.3 Feature selection

We employ a feature selection strategy to pick out the most impactful features from a multitude of options. Specifically, we integrate Principal Component Analysis (PCA) [53] as our chosen dimensionality reduction technique. The aim is to investigate how PCA can contribute to creating more straightforward models, accelerating computational processes, and enhancing the ability to generalize to novel, unseen data. The results are discussed in Subsection 5.6.

### 3.4 Models

Due to the need of personalized model for each patient, and expert domain knowledge by the physiological models and high frequency data generation by the CGM devices, data-driven models have attracted a lot of attention from the research community. As discussed in Section 2, majority of the studies have focused on the Type 1 diabetes and have used the CGM time-series data. T2DM patients’ discrete BGL data has not been used by many previous works. In this paper, we have used and compared the performances of few data-driven models including several machine learning regression models: Support Vector Regression (SVR), Random Forest (RF), and eXtreme Gradient Boosting (XGB), CNN model, and MLP model. We now discuss them in brief as follows.

#### 3.4.1 Random forest (RF)

**Fig. 3:**
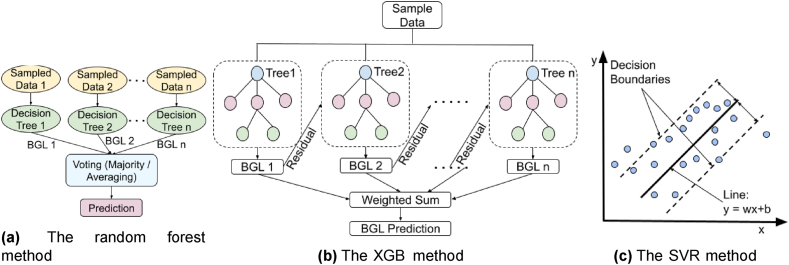
The working principles of the traditional machine learning models used in this study. (a) The random forest method, (b) The XGB method, (c) The SVR method.

*Random Forest* [54] uses a number of decision trees created on various subsets of features randomly selected from the original feature set. Sometimes, more randomness is added by selecting a random threshold for each feature. For regression, it takes the average of the predictions from all the trees (as shown in Fig. 3a). The more the number of decision trees, the better the accuracy, and the better RF handles the overfitting.

#### 3.4.2 Extreme gradient boosting (XGBoost)

*XGBoost* [55] is also a decision tree based sequential learning algorithm that starts with an initial model. The average of all the target values can be used as the predicted value for the initial model. This initial model will have residuals. It then creates subsequent models, where each subsequent model uses a shallow decision tree to fit the residuals from the previous model to correct the errors. Models are added until a fixed number of trees are created, or an acceptable performance is reached, or no more performance improvement is received. The final prediction is the sum of the base model prediction and the weighted sum of all the subsequent models’ predictions (as shown in Fig. 3b). It is a fast and outstanding performing technique for regression problems with structured or tabular data.

#### 3.4.3 Support vector regression (SVR)

Support *Vector Regression* [56] uses the same principles of Support Vector Machine (SVM). SVM is a binary classifier that tries to find a line or hyperplane to separate the two classes well with a max margin between the two decision boundaries. The data points from the two classes are beyond the two decision boundaries. Initially, SVR tries to find a line or hyperplane to separate the two classes well with a good enough margin to have almost all the data points inside the decision boundaries. Then, like *linear regression*, it finds the best fit hyperplane that best fits the data points inside the decision boundaries (as shown in Fig. 3c).

#### 3.4.4 Multi-layer perceptron (MLP)

**Fig. 4:**
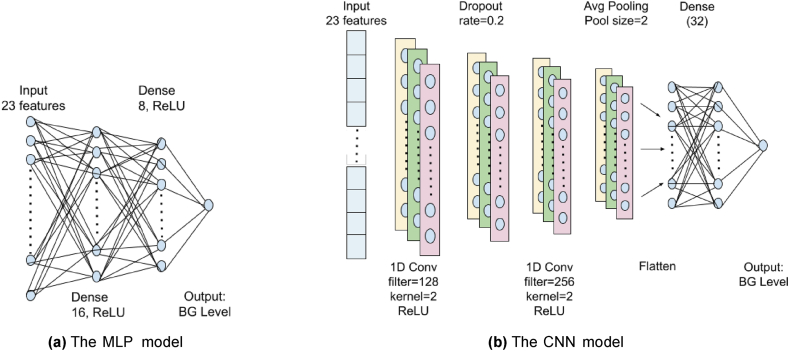
The working principles of the Deep Learning models used in this study. (a) The MLP model, (b) The CNN model.

*Multi-layer Percenptrons* [57] are deep feedforward Artificial Neural Networks (ANNs) where the information flows in one direction from input layer through intermediate hidden computational layers to output layer (as shown in Fig. 4a). All the nodes in one layer are connected with a certain weight to all the nodes in the subsequent layers. All the nodes except the input nodes usually use nonlinear activation functions to control the flow of information to the subsequent layers. These layers are also called *Fully Connected* (FC) dense layers. MLPs use *backpropagation* technique where error in the output is used to improve the network weights. MLPs use training data to train a model to later use it for prediction for new data.

#### 3.4.5 Convolutional neural network (CNN)

Convolutional Neural Networks (CNNs) [58] are ANNs that include convolutional and pooling layers in addition to FC dense layers (as shown in Fig. 4b). CNNs are popularly used for image and speech signal classification and recognition. For a two dimensional (2-D) image, a convolution layer uses a 2-D, for example 3 × 3 kernel filter, which is a 2-D array of weights, to sweep over the entire image starting from top-left corner. Then it finds the dot product of that portion of image and the filter, and slides (shift) right by a few pixels, and repeats this process. The final output is a feature map consisting of the dot product values. The *pooling layers*, like convolution layers, also sweeps across the image with an aggregation function to reduce dimensions and overfitting, improve computational efficiency, and catch global context.

## 4 Experiments

We need to evaluate the candidate models correctly to have a good BGL prediction model. We have applied different ML models as mentioned in Subsection 3.4. Each of the sample data has 23.

features as mentioned in Subsection 3.2. We have conducted a number of experiments to evaluate the models as follows.

Firstly, we have run experiments to find the relationship between the input features and the predicted BGL. We have computed the individual amount of influence of each input feature using information theory concept first. Then, we have used incremental combinations of input features as shown in Table 4 to find the influence of additional information and the best possible combination of features. Here, we have identified our best prediction models. Following this, Principal Component Analysis (PCA) was applied to evaluate its contributions to model performance. The goodness of fit for all models was assessed using R-squared values. Then, we have used one different user's data for testing and other users' data for training. This data separation using different patients is chosen to observe whether the models can generalize. The experimental results are listed in Table 8. The models are then applied to individual patient's data to generate the personalized models. Various split percentages for training and testing data are explored to see whether the optimal split match the already existing consensus. They are listed in Table 10. Additionally, Pearson Coefficients are computed to find the correlation between individual input features and BGL as given in Table 4. This will help to find the most contributing features. Finally, computational and model complexity is computed and compared with previous studies.

**Table 4:** Prediction performances of experiments using incremental input feature combinations in terms of average RMSE values.

Row	Features	CNN	MLP	SVR	Random Forest	XGB
1	Glucose	nan	50.38	51.31	48.86	**48.70**
2	Glucose, Meal	49.76	49.15	50.17	**46.23**	46.51
3	Glucose, Exercise	nan	49.11	48.10	**42.71**	44.31
4	Glucose, Meal, Exercise	47.96	46.44	44.21	**39.22**	41.02
5	Glucose, Meal, Exercise, Med	34.49	33.72	38.78	**32.03**	33.70
6	Glucose, Meal, Exercise, Med, Profile	34.65	33.46	35.35	**31.87**	32.81

For the assessment of all our models, we have split the full dataset into 90% for training data and 10% for testing data. To effectively capitalize our small data set, we have used k-fold cross-validation on the training dataset instead of another one time split into training and validation set of the training dataset. We have tried from 3 folds to 8 folds. Three (3) cross validation folds is found to perform better than others.

The dataset is a 902 × 23 matrix, where 902 is the number of samples, and 23 is the number of input features. For the MLP and CNN models, the first hidden layer takes the 902 × 23 matrix as input.

### 4.1 Evaluation metrics

BGL prediction performance of different models are evaluated in terms of *Root Mean Square*.

*Error* (RMSE) as defined in Equation (2), Clarke's Error Grid Analysis (CEG), R-squared (R2), and.

Adjusted R-squared (R^2^_adj_)

*Root Mean Square Error* (RMSE) is a popular statistical metric in regression analysis. It provides the prediction error of the models. It is widely used and will help us to compare our results with the similar previous studies. Every experiment is run for five times and the average RMSE value is computed and reported here.

(2) $$RMSE=\sqrt{\frac{1}{n}\sum_{1}^{n}{\left(y_{i-}{ŷ}_{i}\right)}^{2}}$$

(3) $$R^{2}=1-\frac{\sum_{1}^{n}{\left(y_{i-}{ŷ}_{i}\right)}^{2}}{\sum_{1}^{n}{\left(y_{i-}\bar{y}\right)}^{2}}$$

(4) $$R_{adj}^{2}=1-\left(1-R^{2}\right)\frac{n-1}{n-k-1}$$

where $y_{i}$ are the actual BGL values, ${ŷ}_{i}$ are the predicted BGL values, $\bar{y}$ is the mean of the actual BGL values, n is the number of data samples, and k is the number of independent variables in the model.

*Clarke's Error Grid Analysis* (CEG) [22] is a clinical regression metric that provides the clinical suitability of a model. CEG has five regions: A, B, C, D, and E. Region A includes correct predictions, while Region B comprises predictions that, while not entirely accurate, do not result in incorrect treatments. Region C consists of predictions that may lead to unnecessary treatments. Region D represents potentially high-risk failures to detect hypo- or hyperglycemic events. Region E includes predictions that may cause confusion in the treatment of hypo- or hyperglycemia.

*R-squared* (R2) (given in Equation (3)) and *Adjusted R-Squared (*R^2^_adj_*)* (given in Equation (4)) are statistical regression metrics. They are more popularly used to measure the goodness of fit of a model. R2 is also called the coefficient of determination and normally ranges from 0 to 1. R^2^_adj_ is used to include the number of parameters, or coefficients, or vectors, or trees to curb the influence of overfitting. It ranges from negative infinity to 1. For both of them, the closer their values to 1 the more the independent variables can explain the variance in the dependent variables.

### 4.2 Hyper-parameter selection

For the *hyper-parameter* selection of all the models in this study, we have used GridSearch [59]. The optimal number of principal components for PCA is determined to be 5. For SVR, the relevant hyperparameters have included the kernel, regularization parameter (C), kernel coefficient (gamma), and epsilon. Due to the extensive hyperparameter space, we have optimized them separately, resulting in optimal values: kernel (rbf), C (2), gamma (scale), and epsilon (0.05).

Random forest, on the other hand, have hyperparameters such as the number of trees, maximum depth of trees, minimum samples split, minimum samples leaf, and maximum features. The optimized values are found to be: n estimators (250), max depth (5), min samples split (35), min samples leaf (2), and max features (’auto’). For XGBoost, hyperparameters have encompassed learning rate, num-ber of trees, maximum depth of a tree, minimum sum of instance weight needed in a child, subsample, and colsample bytree. The optimal hyperparameters are determined as follows: learning rate (0.01), n estimators (175), max depth (4), min child weight (1), subsample (0.7), and colsample bytree (0.6).

The hyperparameters for MLP have included batch size, epochs, optimizer algorithm, learning rate, activation function, and the number of neurons in each layer. After thorough exploration, the optimal settings are identified as: batch size (40), epochs (225), optimizer (Adam), learning rate (0.001), activation function (Relu), and number of neurons (16-8-1). Finally, for CNN, the considered hyperparameters are batch size, epochs, optimizer algorithm, learning rate, activation function, pooling type (max, avg, global), kernel size (2, 3), and number of neurons in each layer. The optimal architecture identified is Conv128-Conv256-Flatten-Dense32-1, with additional optimal hyperparameters: batch size (5), epochs (50), optimizer (adam), learning rate (0.001), activation function (relu), pooling type (avg), and kernel size (2).

## 5 Results and analysis

### 5.1 Assessing the impact of input features on BGL prediction

**Fig. 5:**
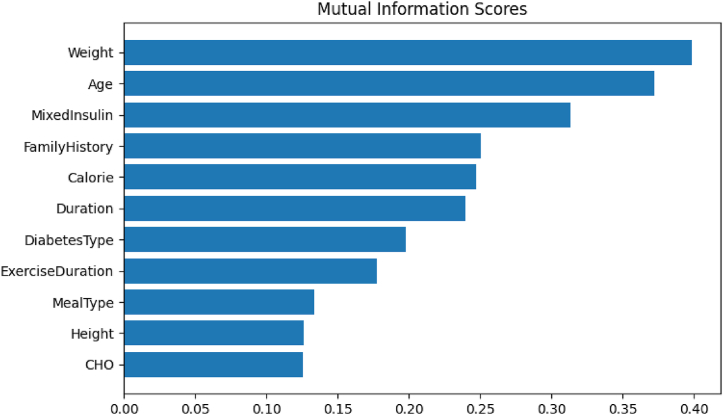
Relationship between significantly contributing input features and BGL.

Different input features have different levels of influences on BGL. Here, we present the analysis on the influence of individual input features, their combinations, and meal macronutrients. To find the most useful features we have computed the Mutual Information (MI) [60], which measures the relationship in terms of uncertainty between a feature and the target variable, for all the input features (shown in Fig. 5). Due to the limited number of subjects in our dataset, the profile features: age, weight, height, diabetes duration, family history and medication features have displayed strong relationship as expected. It is observed that calorie and exercise duration have moderate relationship with BGL.

### 5.2 Comparative analysis of feature combinations in BGL prediction models

We have used different combinations of input features to find the best possible combination of features. The results are shown in Table 4. The performances of the five models are given on separate columns. To observe the influence of additional information, we have incrementally added features starting from glucose (Row 1), then meal & exercise separately (Rows 2 & 3), then three features with both meal & exercise (Row 4), then medicine (Row 5), and finally profile (Row 6). We observe that with each additional feature, the performance has been improved. We observe comparing Rows 1 and.

2, and Rows 3 and 4, that meal information improves performance. Comparing Rows 4 and 5, we observe that medicine improves the performance significantly as expected.

It is also observed that the traditional ML technique random forest provides the best performance with an average RMSE of 40.15 mg/dL over all feature sets. This is because random forest uses decision trees with minimal overfitting. Decision trees usually map input features to output better than other computational methods specially from simple datasets like ours. Therefore, for our carefully designed dataset random forest performs better to map the input features to BGL than other models. The CNN model has come third with an average RMSE of 41.72 mg/dL over all feature sets. It is a good performance considering the limited number of samples. The optimal performance is observed when using glucose, meal, exercise, medicine, and profile as the feature set, employing the random

forest model, resulting in an RMSE value of 31.87 mg/dL. Notably, this achievement closely aligns with the top-performing research outlined in Refs. [11,16]. It's noteworthy that a significant portion of existing research concentrates on predicting short-term horizons, typically around 30 min. This is due to the inherent challenges associated with forecasting blood glucose levels (BGL) over longer intervals, which involve a more extensive array of influencing factors. This is further exemplified in the work by Zarkogianni et al. [11], a prediction model based on Self-Organizing Maps (SOM) and trained on 6 days of CGM readings, changes in CGM readings, and physical activity data from 10.

T1DM patients, that yields an RMSE of 31 mg/dL for a 2-hr PH. Similarly, Karim et al. [16] have proposed a hybrid model trained on 3 weeks of meal, insulin, and CGM readings data from 5 diabetic patients, achieving an RMSE of 31.5 mg/dL for a 2-hr PH. These studies have used continuous CGM data. These comparative benchmarks underscore the competitiveness of our proposed model using discrete data points in the realm of 2-hr BGL prediction.

Moreover, we have computed the CEG prediction percentages as given in Table 5. It is observed that both RFR with a prediction percentage of 98.79% and XGB with a prediction percentage of.

**Table 5:** CEG prediction percentages for the top performing feature group for 2-hr PH.

Model	A	B	A + B	C	D	E
CNN	67.18	31.49	98.67	0.11	1.22	0
MLP	69.62	28.94	98.56	0.22	1.22	0
SVR	67.18	30.38	97.56	0	2.44	0
RFR	71.18	27.61	98.79	0.11	1.11	0
XGB	68.07	31.04	99.11	0.22	0.67	0

99.11% have performed better than or close to the majority of the existing research works [16,19,20,32] in the A + B prediction regions for a 2-hr PH. For instance, Velasco et al. [32] have utilized a dynamic time warping algorithm to segregate the data, have employed Markov Chain Monte Carlo sampling for data augmentation, have trained a Grammatical Evolution (GE) model, and have finally achieved a 99.56% prediction percentage in the A + B regions. Liu et al. [20] have applied a physiological model to two weeks of data from 10 T1DM patients, attaining a 95.27% prediction percentage in the A + B regions. Kushner et al. [19] have used a feed-forward neural network on a dataset of 24 T1DM patients, achieving a 94% correct BGL prediction percentage in the A + B regions. Karim et al. [16] have reported a 98.13% correct BGL prediction percentage in the A + B regions. Among the RFR and XGB models, it is notable that the XGB model has demonstrated superior capability in avoiding potentially hazardous failure to detect hypo- or hyperglycemia events, albeit with a slightly lower percentage in the D region.

### 5.3 Assessing the impact of food macronutrients on BGL prediction

**Fig. 6:**
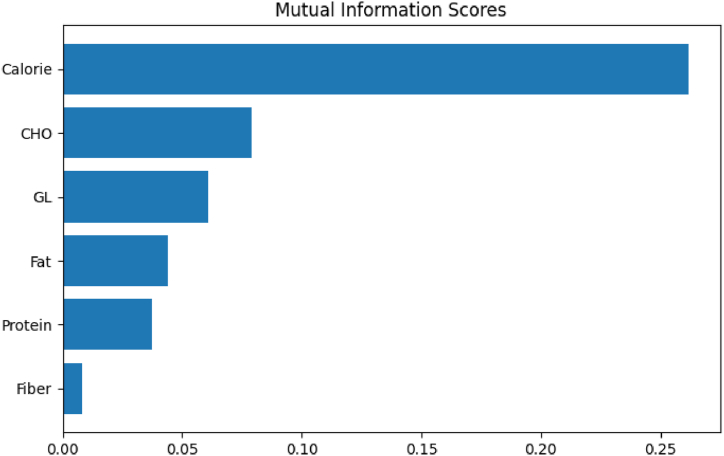
Relationship between the meal macronutrients and BGL.

Meal is considered as one of the key contributors to blood glucose. It is expected that meal intake will increase BGL. Meal information should help predicting the BGL better. As discussed in Subsection 5.2, the impact of meal intake in BGL prediction is moderate in our experiments. We have computed the MI scores of meal macronutrients as displayed in Fig. 6. To investigate further, we have run experiments targeting the meal macronutrient values: CHO, protein, fat, fiber, GL, and calorie. In the feature set for each experiment, in addition to glucose, we have used the meal macronutrients. The performance result is given in Table 6. In one experiment (last row of the table), all the macronutrients are present, and in all other experiments one of the meal macronutrients is absent. For example, in the first row, only the CHO is absent in the feature set, as mentioned by.

**Table 6:** Influence of meal macronutrients reflected by the RMSE values. All feature sets include the BGL measured before meal intake. They (except the last row) also include all the meal macronutrients except one as mentioned in the parentheses.

Feature Set	CNN	MLP	SVR	Random Forest	XGB
Glucose, Meal (except CHO)	47.77	50.65	50.31	46.75	50.92
Glucose, Meal (except Protein)	48.42	50.11	50.13	47.02	52.38
Glucose, Meal (except Fat)	48.46	50.33	50.17	46.76	52.43
Glucose, Meal (except Calorie)	47.53	50.49	50.18	47.03	52.67
Glucose, Meal (except GL)	48.22	50.05	50.13	46.98	52.79
Glucose, Meal (except Fiber)	34.27	34.48	40.52	32.52	35.16
Glucose, Meal	34.06	35.37	35.98	32.61	35.50

”except CHO” in the parentheses. We observe that the best performance is achieved when all the macronutrients are present. Fiber is observed as the only macronutrient with negligible contribution to the performance. For all other macronutrients, the performances are accordingly. That means, the rest of the macronutrients contribute significantly to the prediction.

### 5.4 Statistical significance assessment of BGL prediction models

Regardless of the chosen model, on average the best contributing feature group is Glucose, Meal, Exercise, Medicine, and Profile. For this group of features, we have conducted significance tests to see if one model performs significantly better than the others. In our study, we have found that.

the paired *t*-test is appropriate for comparing two models, such as random forest and CNN. This is because the predictions made by these models can be thought of as paired observations. To ensure the reliability of our comparison, we have first checked if the differences between the paired observations followed a normal distribution. We have used the Shapiro-Wilk test for this purpose. Additionally, we have visually inspected the distribution by creating a histogram. Once we have confirmed that the differences were approximately normally distributed, we have proceeded to perform paired t-tests between the pairs of models. After conducting these tests, we have observed that there is no statistically significant difference in the prediction performance of the random forest, CNN, and XGBoost models. The other two SVR and MLP do have statistically significant difference in the prediction performance.

### 5.5 Fitness assessment of BGL prediction models

The R-squared performance metric serves as a crucial evaluation tool alongside RMSE and CEG, offering insights into the goodness of fit between a model and the sample data. It measures the proportion of the variance in the dependent variable that is predictable from the independent vari-ables. Additionally, adjusted R-squared is computed to enhance the model's generalization capability, mitigating potential overfitting issues.

Upon scrutinizing the adjusted R-squared results, presented in Table 7, it becomes evident that the CNN model exhibits the highest level of compatibility with the dataset. While the CNN performance may marginally lag behind the other four methods, the adjusted R-squared outcomes suggest that it is exceptionally well-suited to capture the underlying patterns within the data. Consequently, despite its slightly inferior performance in comparison to other methods, the CNN model emerges as a favorable choice, emphasizing its robust fit and potential for effective predictions.

**Table 7:** Performances of different models with and without using PCA. All the available 23 features are used. Notations - Adj R2: Adjusted R2, Timet: training time in seconds, Timep: prediction time in seconds. These experiments are run on Google Colab using GPU.

	Without PCA					With PCA				
	CNN	MLP	SVR	RF	XGB	CNN	MLP	SVR	RF	XGB
RMSE	34.65	33.46	35.35	31.87	32.81	36.73	33.90	35.84	33.50	34.10
CEGA + B	98.67	98.56	97.56	98.79	99.11	98.34	98.45	98.12	98.78	98.89
R2	0.53	0.56	0.50	0.59	0.57	0.46	0.54	0.49	0.55	0.54
Adj R2	1.00	−0.13	0.49	0.58	0.56	1.00	0.37	0.48	0.55	0.53
Timet	92.0	218	0.134	1.929	0.467	82.56	216.1	0.94	1.61	0.24
Timep	0.23	0.123	0.002	0.013	0.011	0.289	0.148	0.002	0.034	0.053

**Table 8:** Experiments on measuring the generalizability of the models. Model performances are measured in RMSE values.

Ids, # of Samples	SVR- Train	SVR- Test	RF- Train	RF- Test	XGB- Train	XGB- Test
Train: [7,[10], [11], [12]], 718; Test: [9], 184	41.37	40.76	30.13	39.54	33.29	46.67
Train: [[9], [10], [11], [12]], 717; Test: [7], 185	37.41	86.51	32.34	33.66	36.22	88.49
Train: [7,9,11,12], 732; Test: [10], 170	40.11	48.89	34.40	49.66	38.52	48.94
Train: [7,9,10,12], 716; Test: [11], 186	33.55	52.68	30.33	49.62	32.96	59.86
Train: [7,[9], [10], [11]], 725; Test: [12], 177	43.90	31.54	34.23	35.28	36.93	30.40

The scarcity of studies reporting adjusted R-squared values for blood glucose prediction within a 2-h horizon adds significance to our findings. In one of these limited studies, Bock et al. [23] noted an R-squared value of 0.72 for their model. In comparison, our CNN model has demonstrated a superior R-squared of 1, as detailed in Table 7. While acknowledging the slightly less favorable performance than other models, this study highlights the CNN model's prowess in achieving superior fit and good predictive accuracy.

### 5.6 Impact of PCA-based feature selection on model performance and efficiency

We have used PCA to select a few features from a lot of features to simplify the model. The outcomes, presented in Table 7, indicate a notable reduction in training time for XGB and SVR, a moderate decrease for CNN and RF, and minimal impact for MLP. However, prediction times have slightly increased. Evaluation metrics such as RMSE, CEG, R-squared, and adjusted R-squared have experienced a slight degradation, aligning with our expectations. Consequently, the employment of PCA did not yield significant computational efficiency gains for all the models, especially for our best performing random forest model.

### 5.7 Generalizability of the models across different data-distributions

In this subsection, we have run experiments to present the generalizability of the proposed data-driven models. In these experiments, training and testing data comes from different distributions, i.e., one user's data is used for testing and other users' data are used for training. The results are listed in Table 8. In our collected dataset, we have five subjects' data. In the model training, data of four subjects are used as training data with Cross Validation (CV). The results (RMSE values) are given in the -Train columns. For model testing, the remaining subject's data is used as the test data. The results (RMSE values) are given in the -Test columns. The RMSE values of CNN, and MLP models prediction result on test data are below satisfactory. This is due to the small size of the dataset, and non-linear nature of the data. SVR, random forest, and XGB models have reasonably good RMSE values. Hence, these three models can generalize well. RF model has the best performance as given in Table 8. We observe that for RF the test results are closest to the cross validation results.

### 5.8 Prediction performance analysis of the personalized models

Personalized models have been generated by applying the modeling techniques on individual patient data. The results are given in Table 9. The models trained with data of patient ids 10 and 12 observe best results with low average RMSE values of 19.53 mg/dL and 25.85 mg/dL. This is due to the fact that the patient with Id 10 has been recently diagnosed with diabetes. The patient with Id 12 is prediabetic. The differences between their before meal and after meal BGLs have low variance. On the other hand, other patients have a long history of diabetes. The differences between their before meal and after meal BGLs have high variance.

**Table 9:** Personalized model performances (evaluated in RMSE values).

Experiments	CNN	MLP	SVR	Random Forest	XGB
Id: 7, Samples: 185	35.83	33.86	37.27	33.32	32.28
Id: 9, Samples: 184	41.23	40.31	46.49	39.18	40.54
Id: 10, Samples: 170	20.54	21.78	23.31	19.38	19.16
Id: 11, Samples: 186	43.69	39.80	42.98	39.35	36.93
Id: 12, Samples: 177	24.09	24.11	29.14	22.64	22.55

**Table 10:** Model performances of the train-Test split experiments measured in RMSE values.

Experiments	CNN	MLP	SVR	RF	XGB
Train:10%, Test:90%	57.79	44.06	48.15	37.94	40.17
Train:20%, Test:80%	39.75	37.39	41.79	34.67	38.96
Train:30%, Test:70%	37.33	36.96	40.44	32.78	36.48
Train:40%, Test:60%	34.80	34.77	38.29	32.23	35.67
Train:50%, Test:50%	34.10	36.00	36.40	31.38	34.11
Train:60%, Test:40%	33.55	32.83	34.56	30.69	33.52
**Train:70%, Test:30%**	**31.79**	**32.69**	**33.03**	**30.08**	**31.26**
Train:80%, Test:20%	33.43	33.5	34.65	32.05	31.73
Train:90%, Test:10%	33.23	32.5	33.80	32.71	31.13

### 5.9 Identifying the optimal training-testing data split

We have run experiments with various split percentages for training and testing data. They are listed in Table 10. With the increase of training data, performances of the models improve, and the RMSE decreases. This happens up to the split percentage of training: 70.0%, testing: 30.0%. After that, with an increase in training data, the performance gets worse. This is due to the overfitting of the data by the models. Therefore, training: 70.0%, testing: 30.0% data split brings the best performance. This result from our discrete value dataset matches the already existing consensus in the research community.

### 5.10 Challenges in finding correlations between single features and BGL

*Pearson coefficients* are computed from raw data between individual input feature and 2 h after meal BGL as given in Table 11. The macronutrients and other characteristics (CHO, protein, fat, fiber, calorie, and GL) of food contribute differently to the BGL. BGL increases with an increase of CHO. Body takes more time to break down protein. And protein doesn't contribute directly to BGL. Hence, with more protein BGL may be less within a certain time. Similarly, due to slow digestion, BGL may be less with an increase of fiber in food within a certain time. Though fat does not have any direct influence on BGL, high fat causes less digestion. In turn this makes insulin become less effective. This might cause BGL to increase with fat increase. In addition to food macronutrients, physical exercise, medication, and many other factors influence BGL. Therefore, it would be difficult to see the one to one relationship as mentioned above. That is what is observed from the pearson coefficients of different subjects (with IDs 7, 9, 10, 11, and 12) in Table 11. For example, for the patient with ID 10 (see the column with ID 10), for exercise duration, we observe a positive correlation of.

**Table 11:** Pearson coefficients between BGL contributors and 2 h after meal BGL.

BGL Contributors	7	9	10	11	12
Exercise Duration	0.097	0.025	0.234	−0.250	−0.032
CHO	0.090	0.0602	−0.192	0.013	0.024
Protein	0.131	−0.083	−0.174	0.014	0.005
Fat	−0.072	0.036	−0.169	0.143	0.034
Fiber	−0.054	−0.090	−0.144	0.055	0.063
Calorie	0.052	0.034	−0.226	0.120	0.050
GL	0.051	0.057	−0.321	0.093	0.018

0.234 though theoretically it has a negative correlation with BGL; for CHO, we observe a negative correlation of −0.192 though theoretically it has a positive correlation with BGL; etc.

### 5.11 Simplicity and efficiency analysis of the BGL prediction models

The computational and model complexities of the models employed in this study are detailed in Table 12, with their corresponding hyperparameters provided in subsection 4.2. All of our models demonstrate simplicity, as evident from both the training and prediction times presented in the ta-ble. These computations are executed on a laptop PC equipped with an Intel Core i5-9300H CPU.

**Table 12:** Computational and model complexity of different models used in this study. *Timetrain* and *Timeprediction* are training and prediction time in seconds. *Timetrain* and *Timeprediction* complexities given below are simplified approximations based on the common operations of the methods. Notations - n: number of samples in the training data, f: number of features/channels, t: number of trees, m: avg. # of neurons in each layer, l: # of layers, h: height of input channel, w: width of input channel, c: # of filters, k: size of Conv kernel, P: parameters, SV: Support Vectors, BR: Boosting Rounds.

	CNN	MLP	SVR	RF	XGB
*T ime*_*train*_ Complexity	O(nfhwc*k*2l)	O(nmfl)	O(*n*2f)	O(nflogn)	O(tnflogn)
*T ime*_*train*_	85.16	100.03	0.26	2.58	0.6
*T ime*_*prediction*_ Complexity	O(fhwc*k*2l)	O(mfl)	O(f)	O(tlogn)	O(tlogn)
*T ime*_*prediction*_	0.387	0.281	0.001	0.031	0.016
Model complexity	P: 156353	P: 433	SV: 718	t: 250	BR: 175

@ 2.40 GHz and 24.0 GB of installed RAM. Notably, CNN stands out as one of the slower models, requiring just over a minute for training and 387 ms for inference. Despite this, their per-formance aligns with existing works [6,61] focusing on online model retraining, and with previous studies [6,7,38,[61], [62], [63]] focusing on online prediction. This ensures their suitability for deployment on smart devices for predictive purposes.

## 6 Discussion

In this section, we have presented the main outcomes of this study, outlined the study's limitations, and potential directions for future research. These aspects are described in detail below.

### 6.1 Key findings

In this study, we observed that different input features exert varying influences on Blood Glucose Level (BGL). The analysis explores individual features, their combinations, and meal macronutrients. Mutual Information (MI) is computed to measure the relationship between each feature and the tar-get variable. Profile features show a strong relationship, while calorie and exercise duration exhibit a moderate connection with BGL. Meal macronutrients, especially when combined with glucose, sig-nificantly contribute to BGL prediction. Experiments targeting macronutrient values reveal that the presence of all macronutrients leads to the best performance, with fiber being the only macronutrient showing negligible contribution.

Various input feature combinations are assessed for optimal performance. The results, displayed in Table 4, reveal the effectiveness of different models. Random forest demonstrates the best performance with an average Root Mean Square Error (RMSE) of 40.15 mg/dL. Incrementally adding features im-proves performance, and the best results are achieved with the combination of glucose, meal, exercise, medicine, and profile, employing the random forest model with an RMSE value of 31.87 mg/dL. Signif-icantly, this accomplishment closely aligns with the top-performing research highlighted in Refs. [11,16] The study acknowledges the challenges associated with predicting blood glucose levels over longer inter-vals, contrasting with existing research that often focuses on shorter-term horizons. Paired t-tests are conducted to compare model performances, particularly between random forest, CNN, and XGBoost. The analysis confirms no statistically significant difference among these models. However, SVR and MLP exhibit significant differences in prediction performance. Additionally, Cumulative Error Grid (CEG) prediction percentages highlight the competitive performance of random forest and XGBoost models.

Principal Component Analysis (PCA) is employed to simplify models, with outcomes indicating reduced training time for some models, despite a slight increase in prediction times and minor degra-dation in evaluation metrics (RMSE, CEG, R-squared, adjusted R-squared). Model fitness is assessed using R-squared values. The CNN model exhibits the highest compatibility, emphasizing its robust fit and potential for effective predictions within the given context.

Experiments on model generalizability, where training and testing data come from different dis-tributions, reveal reasonable performance for SVR, random forest, and XGB models. Random forest demonstrates the best performance in this context. Personalized models, generated from individual patient data, show varying performance. Patients with recent diabetes diagnoses or prediabetic con-ditions exhibit lower variance in before and after meal BGLs, resulting in better model performance. The computational and model complexities of the employed models are detailed. Despite the slower training time of the CNN and MLP models, all models demonstrate simplicity and suitability for deployment on smart devices for predictive purposes.

### 6.2 Limitations and future works

From the results, we can see that our best generalized models provide a state of the art or near state of the art performances. Yet, they have the following limitations.

1. *Small Sample Size*: The limited dataset, comprising information from only 5 patients, limits the generalizability of the developed models. To enhance robustness and applicability across a broader population, acquiring more data from diverse patients is crucial.
2. *Limited Clinical Interpretability*: The use of black-box machine learning models introduces a limitation in terms of clinical interpretability. Their adoption in clinical settings where inter-pretability is crucial can be challenging. Future efforts should aim to improve the transparency of the models.
3. *Prospective Real-World Testing*: While the study provides insights into model performance based on historical data, a limitation lies in the absence of prospective testing in real-world scenarios. Evaluating the models in real-time and real-world conditions is essential to validate their practi-cal utility and reliability in diverse clinical settings. Future research should prioritize prospective testing to ensure the models' effectiveness in real-world applications.

Other future research directions can include the integration of additional regression predictive models, expanding the set of input features, implementing data augmentation techniques to address data scarcity issues, offering confidence levels alongside blood glucose level values, and further refining feature engineering approaches.

## 7 Conclusions

Two hours after meal BGL prediction is crucial for T2DM patients as it guides a patient to follow a healthy diet or to avoid any adverse glucose situation. We have made several contributions in this regard. Firstly, we have introduced a 2 h after meal BGL prediction framework from discrete BGL readings of T2DM patients and other parameters such as food intake, medicine, exercise, etc using data-driven models. To the best of our knowledge, this is the first work using data-driven models to demonstrate that discrete BGL values are able to provide a state of the art performance in predicting BGL values. Secondly, we have meticulously designed and collected a unique dataset encompassing discrete BGL values (two per day, one before meal, and one 2 h after meal), medication doses, meal images, physical activities, and profile from five T2DM patients over a six-month period. Thirdly, we have conducted a comprehensive evaluation of several traditional ML regression and Deep Learning techniques using the popular metrics RMSE, CEG, R-squared, and adjusted R-squared values. The random forest model has exhibited the best overall performance, closely trailed by the CNN model. Additionally, we have analyzed the scale of contributions of the input features, the generalization capability of the different data-driven models, and the performances of the personalized models. While the generalized models exhibited reasonable performance across diverse user distributions, personalized models showcased varied results, particularly excelling in cases of recently diagnosed and prediabetic patients. Though some results are not conclusive, we believe that the use of discrete BGL values is promising in predicting 2 h after meal BGLs. We have acknowledged a few limitations and suggested potential avenues for future research of our proposed framework. We emphasize that more research works need to focus on BGL prediction using discrete data from a large cohort of T2DM patients.

In conclusion, our work not only addresses a critical aspect of T2DM management but also con-tributes novel insights into the potential of discrete BGL values and data-driven models. The path forward involves addressing identified limitations and expanding the scope of research to encompass a more extensive cohort, advancing our understanding of predictive modeling in the context of post-meal BGLs for improved diabetes management.

## Ethical approval and consents

Appropriate approval related to data collection involving human subjects is taken from the Ethics Committee of the Bangladesh University of Engineering and Technology (BUET), reference no: Estt./Ta- 4/140548/R-3617, dated 04/12/22. Written Informed Consents have been taken from all the pa-tients/participants to participate and to publish the data of patients/participants as a standard prac-tice of data collection.

## Funding information

This work is part of a research project funded by the Institute for Advanced Research (IAR) at United International University, Grant/Award Number: UIU/IAR/01/2021/SE/11.

## Data availability statement

The dataset used in this study is publicly available at http://tinyurl.com/2bjfqfqw

## CRediT authorship contribution statement

**Benzir Md Ahmed:** Writing – review & editing, Software, Resources, Methodology, Investigation, Funding acquisition, Formal analysis, Data curation, Conceptualization, Validation, Visualization, Writing – original draft. **Mohammed Eunus Ali:** Writing – review & editing, Writing – original draft, Visualization, Supervision, Software, Investigation, Formal analysis, Conceptualization, Methodology, Project administration, Resources. **Mohammad Mehedy Masud:** Writing – review & editing, Writing – original draft, Visualization, Supervision, Project administration, Methodology, Investigation, Formal analysis, Conceptualization. **Mohammad Raihan Azad:** Validation. **Mahmuda Naznin:** Writing – review & editing, Writing – original draft, Supervision, Project administration, Methodology, Investigation, Formal analysis, Conceptualization, Visualization.

## Declaration of competing interest

The authors declare that they have no known competing financial interests or personal relationships that could have appeared to influence the work reported in this paper.
